# Biophysical and biochemical signatures of pancreatic stellate cell activation: insights into mechano-metabolic signalling from atomic force microscopy and Raman spectroscopy

**DOI:** 10.1186/s12964-025-02354-1

**Published:** 2025-08-04

**Authors:** Jacek J. Litewka, Monika A. Jakubowska, Marta Targosz-Korecka, Ewelina Wiercigroch, Jakub Dybas, Natalia Cisak, Zbigniew Madeja, Pawel E. Ferdek

**Affiliations:** 1 Department of Cell Biology, Faculty of Biochemistry, Biophysics and Biotechnology, Jagiellonian University, ul. Gronostajowa 7, Krakow, 30-387 Poland; 2 Doctoral School of Exact and Biological Sciences, Jagiellonian University, ul. Łojasiewicza 11, Kraków, 30-348 Poland; 3 Malopolska Centre of Biotechnology, Jagiellonian University, ul. Gronostajowa 7A, Krakow, 30-387 Poland; 4 Department of Physics of Nanostructures and Nanotechnology, Faculty of Physics, Astronomy and Applied Computer Science, Jagiellonian University, ul. Łojasiewicza 11, Kraków, 30-348 Poland; 5 Jagiellonian Center of Innovation, ul. Bobrzyńskiego 14, Krakow, 30-348 Poland; 6 Jagiellonian Centre for Experimental Therapeutics, Jagiellonian University, ul. Bobrzyńskiego 14, Krakow, 30-348 Poland

**Keywords:** Pancreatic stellate cells, Fibrosis, Lipid metabolism, Cytoskeletal remodelling, Mechanical properties, Raman spectroscopy, Atomic force microscopy, Metabolic reprogramming

## Abstract

**Background:**

Pancreatic fibrosis is a key pathological feature of chronic pancreatitis and pancreatic cancer, driven by the persistent activation of pancreatic stellate cells. These cells, normally quiescent, undergo profound phenotypic changes in response to environmental cues, yet the interplay between mechanical forces and metabolic reprogramming during this transition remains poorly understood. As the stromal microenvironment actively communicates with epithelial and vascular compartments, understanding this mechano-metabolic signalling axis is critical for uncovering novel mechanisms of tissue remodelling.

**Methods:**

To investigate the biomechanical and biochemical alterations during stellate cell activation, we employed atomic force microscopy and Raman spectroscopy to measure changes in cell stiffness, morphology, and molecular composition. These data were complemented by transcriptomic analyses to evaluate gene expression profiles related to lipid metabolism and autophagy. Quantitative statistical tests, including ANOVA and Kruskal-Wallis tests with appropriate post hoc corrections, were applied.

**Results:**

Activation of human pancreatic stellate cells led to progressive cytoskeletal remodelling, increased cellular stiffness, and a flattened morphology. Raman spectroscopy revealed an expansion of the cytoplasmic area, changes in nucleic acid signal, and significant increases in lipid content, particularly in unsaturated lipids and triacylglycerols. Gene expression analysis demonstrated upregulation of lipid elongation and desaturation pathways, along with enhanced autophagy, suggesting a coordinated metabolic adaptation. These changes support the myofibroblast-like phenotype and may influence intercellular signalling by altering extracellular matrix composition, mechanical tension, and the release of signalling molecules that affect the surrounding microenvironment.

**Conclusions:**

Our findings reveal that pancreatic stellate cell activation involves a tightly coupled shift in mechanical and metabolic states, highlighting an integrated signalling process that may modulate stromal–vascular and stromal–epithelial communication. This mechano-metabolic axis represents a potential therapeutic target in fibrotic and neoplastic pancreatic diseases, where aberrant stromal signalling contributes to disease progression.

## Introduction

In recent years, pancreatic stellate cells (PSCs) have attracted growing attention due to their central role in the pathogenesis of pancreatic diseases, including chronic pancreatitis and pancreatic cancer, both associated with high morbidity and limited treatment options [1–3]. It is now well established that PSCs possess the capacity to transition from a quiescent fibroblast-like state to an activated myofibroblast-like phenotype that drives fibrosis and profoundly alters the pancreatic microenvironment [4–6].

In a healthy pancreas, PSCs are typically quiescent, characterised by low metabolic activity and the presence of cytoplasmic vitamin A-containing lipid droplets [4, 7, 8]. This feature suggests that lipid metabolism may play a crucial role in maintaining PSC homeostasis. Quiescent PSCs are believed to contribute to tissue integrity and support pancreatic architecture by forming a matrix that ensures mechanical stability and facilitates tissue repair. Upon pathological stimuli – including chronic inflammation, oxidative stress, mechanical strain, or exposure to various growth factors – PSCs lose their retinoid-containing lipid droplets and acquire an activated phenotype [9, 10]. This transition leads to increased synthesis of extracellular matrix (ECM) proteins and the secretion of pro-inflammatory and pro-fibrogenic factors, notably transforming growth factor β (TGF-β), which reinforces activation through autocrine signalling loops [1, 4, 5, 11, 12].

In addition to chemical signalling, mechanical forces are now recognised as potent regulators of PSC behaviour. Increased tissue stiffness and mechanical stress, typical features of fibrotic pancreatic tissue, can directly activate PSCs and promote their transition towards a contractile, matrix-remodelling phenotype [9, 13]. These changes are not merely a consequence of fibrosis but they actively contribute to disease progression by modulating local signalling dynamics. A hallmark of PSC activation is the upregulation of α-smooth muscle actin (α-SMA), reflecting cytoskeletal reorganisation and altered cellular biomechanics [4, 5]. This suggests that PSCs undergo coordinated biophysical and biochemical reprogramming, which may influence how they interact with their environment.

The consequences of PSC activation extend beyond ECM deposition. Excessive matrix production and cytoskeletal contraction lead to mechanical compression of surrounding structures, including ducts and blood vessels, which impairs perfusion, promotes hypoxia, and contributes to a fibrotic and hypovascular microenvironment [14]. At the same time, activated PSCs release soluble factors such as vascular endothelial growth factor (VEGF), promoting endothelial proliferation, migration, and neovascular signalling [15]. Experimental models have shown that PSC-derived factors, particularly under hypoxic conditions, enhance endothelial responses in vitro and in vivo [15]. Together, these observations position PSCs not only as mediators of fibrosis but also as active participants in stromal –vascular communication, shaping the tissue signalling landscape during pancreatic disease progression.

Despite growing evidence of the involvement of PSCs in mechanical and paracrine signalling, the precise nature of the biophysical and metabolic changes underlying their activation remains poorly defined. In this study, we sought to address this gap by applying two complementary techniques – atomic force microscopy (AFM) and Raman spectroscopy (RS) – to systematically characterise the mechanical properties and molecular composition of quiescent and activated human PSCs. AFM enables quantitative mapping of cellular stiffness and morphology, while RS provides detailed spectral information on biomolecular profiles, including proteins, lipids, and nucleic acids. By integrating these approaches with transcriptomic analysis, we aim to elucidate how PSC activation orchestrates a mechano-metabolic shift that may influence intercellular signalling and microenvironmental remodelling in the fibrotic diseases of the pancreas.

## Materials and methods

### Materials and reagents

Details of the key reagents and resources employed throughout this study are summarised in Table 1.

**Table 1:** Key reagents and resources used in this study. This table summarises the main materials, reagents, antibodies, cell lines, and software used for the experiments described, including their sources and catalogue numbers where applicable

REAGENT or RESOURCE	SOURCE	IDENTIFIER
Antibodies		
mouse anti-α-SMA	Abcam	ab7817
rabbit anti-vimentin	Thermo Fisher Scientific	MA5-35320
goat anti-mouse Alexa Fluor 488 antibody	Thermo Fisher Scientific	A-11001
goat anti-rabbit Alexa Fluor 635 antibody	Thermo Fisher Scientific	A-31576
**Chemicals**,** peptides**,** and recombinant proteins**		
Cytochalasin D	Thermo Fisher Scientific	PHZ1063
Human TGF-β	Corning	354039
ProLong Diamond Antifade Mountant with DAPI	Thermo Fisher Scientific	P36962
Fenozol Plus	A&A Biotechnology	203-100P
TRIzol™ Reagent	Thermo Fisher Scientific	15596026
**Critical commercial assays**		
High Capacity cDNA Reverse Transcription Kit	Applied Biosystems	4368814
GoTaq^®^ qPCR Master Mix	Promega	A6002
Total RNA Mini Kit	A&A Biotechnology	031–100
PureLink RNA Mini Kit	Thermo Fisher Scientific	12183020
**Experimental models: Cell lines**		
Human Pancreatic Stellate Cells	ScienCell	3830
Stellate Cell Medium	ScienCell	5301
**Oligonucleotides**		
Forward primer for ***ACTA2*** (α-SMA, alpha smooth muscle actin): ACTGCCTTGGTGTGTGACAA	Genomed	N/A
Reverse primer for ***ACTA2*** (α-SMA, alpha smooth muscle actin): CACCATCACCCCCTGATGTC	Genomed	N/A
Forward primer for ***VIM*** (vimentin): AAATGGCTCGTCACCTTCGT	Genomed	N/A
Reverse primer for ***VIM*** (vimentin): AGAAATCCTGCTCTCCTCGC	Genomed	N/A
Forward primer for ***RPL28*** (60 S ribosomal protein L28): GACCTACAGCACTGAGCCCAATAAC	Genomed	N/A
Reverse primer for ***RPL28*** (60 S ribosomal protein L28): TGGTGGTCCGCACATAGGA	Genomed	N/A
Forward primer for ***EEF2*** (eukaryotic elongation factor 2): GACATCACCAAGGGTGTGCAG	Genomed	N/A
Reverse primer for ***EEF2*** (eukaryotic elongation factor 2): TTCAGCACACTGGCATAGAGGC	Genomed	N/A
**Software and algorithms**		
GraphPad Prism 10	Graphpad Software	https://www.graphpad.com/
DESeq2 package	Michael I Love et al.	https://bioconductor.org/packages/release/bioc/html/DESeq2.html
Cutadapt	Marcel Martin	https://cutadapt.readthedocs.io/en/stable/
FASTQC	Simon Andrews	https://www.bioinformatics.babraham.ac.uk/projects/fastqc/
HISAT2	Yun Zhang et al.	https://daehwankimlab.github.io/hisat2/
**Other**		
MicroAmp™ Fast Optical 96-Well Reaction Plate	Applied Biosystems	4346907
The Human Protein Atlas	The Human Protein Atlas	https://www.proteinatlas.org/

### Cell culture

Human pancreatic stellate cells and specialised stellate cell medium (SteCM) with foetal bovine serum (FBS) at concentration 2%, cell growth supplements (CGS) and penicillin/streptomycin (P/S) solution were obtained from ScienCell, Carlsbad, CA, USA. The PSCs were cultured in complete SteCM in T25 flasks at 37 °C in a 5% CO_2_ atmosphere and passaged weekly according to predetermined procedures [8]. To induce activation of PSCs, we used a previously developed protocol [12]. Briefly, the cells were cultured in ‘incomplete’ SteCM medium, lacking FBS and CGS, but were supplemented with 5 ng/ml TGF-β (Corning, New York, NY, USA) for either 2, 5 or 7 days, with medium changes every 3–4 days. Spontaneous activation was achieved only by culturing cells in incomplete SteCM medium for 2 days. Regular PCR-based assays were performed to verify potential mycoplasma infection.

### Immunocytochemistry

For immunocytochemical staining, PSCs were seeded onto Ø13 mm round glass coverslips placed in 24-well plates. After incubation, the cells were washed with phosphate-buffered saline (PBS) and fixed and permeabilised with 100% methanol at -20 °C for 20 min. To block non-specific binding, cells were incubated with 2% bovine serum albumin (BSA) in PBS for 60 min at RT, followed by incubation with primary antibodies: mouse anti-α-SMA (Abcam, Cambridge, UK) and rabbit anti-vimentin (Abcam) diluted 1:300 in 1% BSA for 2 h at RT. After washing 3 times in PBS, cells were incubated with the secondary goat anti-mouse Alexa Fluor 488 antibody (Thermo Fisher Scientific, Waltham, MA, USA) and goat anti-rabbit Alexa Fluor 635 antibody (Thermo Fisher Scientific) diluted 1:500 in 1% BSA for 1 h at RT. Cells were subsequently washed with PBS (4 × 5 min) and mounted on microscope slides using ProLong Diamond Antifade Mountant with DAPI (Thermo Fisher Scientific). Imaging was performed using a Leica DMi8 fluorescence microscope (Leica Microsystems, Wetzlar, Germany) equipped with a DFC7000GT camera and an HC PL APO 40x/1.30 OIL objective. Imaging parameters were 365 nm (DAPI) 200 ms, 490 nm (FITC) 300 ms, and 635 nm (CY5) 300 ms, each with 3–4% illumination power with binning 2 × 2 (960 × 720 px). A minimum of ten representative images were taken for each condition.

### Atomic force microscopy

For AFM measurements, 50,000 PSCs were seeded onto 60-mm plastic Petri dishes (Sarstedt AG & Co. KG, Nümbrecht, Germany, cat. no. 83.3901). The cells were maintained in either their quiescent phenotype or activated state, as previously described. Prior to the experiments, the culture medium was replaced with HBSS buffer. AFM measurements were performed with a Bruker JPK NanoWizard3 microscope (JPK Instruments, Berlin, Germany). All samples were tested in liquid isotonic electrolyte conditions (HBSS, Thermo Fisher Scientific) at room temperature (checked before each measurement and included as a calibration parameter, ~ 22 °C). Two modalities of AFM microscopy operation were used to obtain a quantitative analysis of the elastic modulus and a qualitative analysis of the topography of the measured cells, correlated with a graphical representation of the elastic modulus distribution in the cell (high-resolution 2-dimensional elasticity maps).

To investigate the role of the actin cytoskeleton in determining the mechanical properties of PSCs, cells were treated with 50 µM cytochalasin D (Cyt D, Merck, Darmstadt, Germany; stock solution prepared in DMSO). The working concentration was diluted in HBSS buffer. Cells of all phenotypes (qPSC, sPSC, 2dPSC, 7dPSC) were incubated with Cyt D for up to 60 min. To assess time-dependent effects, AFM measurements were performed immediately prior to the addition of Cyt D (0 min), and subsequently after 15, 30, 45, and 60 min of treatment. A parallel set of samples was fixed and stained with phalloidin at 30 min to visualise actin filament integrity via fluorescence microscopy.

The force mapping mode was used to determine the elastic modulus of the PSCs. A Bruker MLCT-BIO-DC type C cantilever with a quadratic pyramid probe was used for this method. For each sample, at least 10 independent force maps were acquired, each covering an area of 40 × 40 μm, with a preserved scaling of 16 px / 10 μm. A force-distance curve was plotted in each px. More than one cell was measured in the area of one map; a total of 20–23 cells were measured for one sample, with at least 16 force-distance curves for one cell. A constant indentation range, not exceeding 300 nm, was maintained throughout the measurements to avoid the influence of the hard substrate on the resulting Young’s modulus values. The set of force-distance curves was analysed by fitting the Hertz-Sneddon model for a pyramidal indenter to determine the cell modulus E (which refers exclusively to the apparent Young’s modulus). All analyses were performed using JPK data processing software.

Topography images correlated with high-resolution elastic maps were acquired using a selected force-distance (FD)-based imaging mode (Quantitative Imaging, QI; JPK Instruments). In this method, a single FD curve is measured at each pixel point of the image and translated from the selected trigger force into the cell topography images. The QI measurement was performed using conical Pt-Ir coated cantilevers (SCM-PIC-V2, Bruker) with a nominal spring constant of 0.1 N/m. Images (256 × 256 pixels) were obtained with a scan size of 40 × 40 μm. The loading force varied from 0.6 to 1.0 nN (indentation depth between 300 and 500 nm) and was adjusted to obtain a clear contrast of the cell surface. To determine the elastic modulus map, the set of force-distance curves was analysed by fitting the Hertz-Sneddon model for a conical indenter. The topography analysis (height of cells) and elastic modulus images were derived using JPK data processing software.

To reinforce the analysis of cell height, the Root Mean Square (RMS) roughness was calculated as a measure of deviations of the cell surface profile from its mean line. This parameter was determined using ten surface cross-sections along the scan line in the central region of the cell. Selecting the central area minimised the influence of overall cell curvature and enabled the assessment of local topographical variations. The analysis was performed on raw data from high-resolution height maps using JPK data processing software.

### Raman spectroscopy

For RS, cells were passaged 24 h prior to measurement and seeded on calcium fluoride (CaF_2_) microscope slides. Before imaging, the culture medium was replaced with NaHEPES buffer (140 mM NaCl, 4.7 mM KCl, 10 mM HEPES, 1 mM MgCl_2_, 10 mM glucose; pH 7.2; supplemented with 1 mM CaCl_2_) [8]. Raman imaging was performed using a WITec confocal Raman microscope (WITec 300R, Ulm, Germany) equipped with a CCD detector cooled to − 60 °C and using a 532 nm excitation wavelength. The laser was coupled to the microscope through a 50 μm optical fibre, and the spectral resolution was approximately 4 cm^− 1^. The monochromator was calibrated using a xenon lamp (WITec UV light source), and single-point calibration was performed with the Raman scattering line of a silicon plate (521 cm^− 1^) before each measurement session.

For each experimental group, Raman images of 30 cells were acquired using a water-immersive Zeiss W-Plan Apochromat 63×/1.00 W objective, with a 2 μm sampling density in the x/y direction, 0.5 s integration time, and 25 mW laser power. In addition, higher-resolution images of 9 cells per group were acquired with a 0.5 μm sampling density in the x/y direction and a 0.2 s integration time to enhance the differentiation of cellular compartments.

Raman data analysis was performed using WITec Project Plus 5.30 (WITec GmbH, Ulm, Germany) and OriginPro 2022 software (OriginLab Corporation, Northampton, MA, USA). Raman images were generated through integration analysis of specific marker bands (detailed in figure captions) or by performing k-means cluster analysis (KMC).

### RNA isolation, reverse transcription, and real-time PCR

Total RNA was extracted from PSCs of different phenotypes cultured in 6-well plates using the Fenozol Plus and Total RNA Mini Kit (A&A Biotechnology, Gdynia, Poland) according to the manufacturer’s protocol. RNA concentrations were measured using a NanoDrop™ 2000 spectrophotometer (Thermo Fisher Scientific). For cDNA synthesis, 1 µg of total RNA was used with the High Capacity cDNA Reverse Transcription Kit (Thermo Fisher Scientific), following the manufacturer’s instructions. Real-time PCR was performed using GoTaq^®^ qPCR Master Mix (Promega, Madison, WI, USA) and specific gene primers (Genomed, Warsaw, Poland) (Table 1), using a MicroAmp™ Fast Optical 96-Well Reaction Plate (Applied Biosystems, Waltham, MA, USA). RT-PCR assays were run on the QuantStudio™ 12 K Flex Real-Time PCR System (Applied Biosystems) under the following conditions: 50 °C for 20 s and 95 °C for 5 min, followed by 40 cycles of 95 °C for 15 s and 60 °C for 1 min. The relative gene expression was determined using the cycle threshold (Ct) value, normalised to the geometric mean of two reference genes: 60 S ribosomal protein L28 (*RPL28*) and eukaryotic elongation factor 2 (*EEF2*) using the 2^(−∆∆Ct)^ method [16].

### RNA isolation and sequencing

For sequencing, total RNA was extracted from PSCs of different phenotypes cultured in 6-well plates using the PureLink RNA Mini Kit (Thermo Fisher Scientific, Waltham, MA, USA) with the TRIzol option. The RNA was subsequently treated with DNase to eliminate residual DNA contamination. The isolated RNA was of high quality, characterised as total RNA free of protein contamination, and suspended in RNase-free water. Each sample had a minimum quantity of 2 µg, a volume of at least 20 µl, and a concentration exceeding 40 ng/µl. The purity of the RNA was confirmed with an OD260/280 ratio ranging between 2.0 and 2.2, while RNA integrity was ensured with a 28 S:18 S ratio of at least 1.0 and an RNA Integrity Number (RIN) equal to or greater than 7.0.

After isolation, RNA libraries were prepared and sequenced to generate paired-end reads. The sequencing was performed by an external company, Genomed (Warsaw, Poland), using an Illumina sequencing platform. Following sequencing, the raw reads were processed by trimming adapter sequences using Cutadapt with a quality threshold of q = 25 and a minimum read length of 20 nucleotides. The quality of the trimmed reads was evaluated using FASTQC.

Reads were then aligned to the human reference genome (GRCh38.p14) using HISAT2, with strand-specific library preparation (–rna-strandness RF) enabled. Mapping quality was assessed, and reads were quantified using HTSeq, counting reads on the reverse strand to produce gene-level expression counts. Gene annotations were sourced from a feature table based on the GRCh38 reference.

The differential expression analysis was conducted using the DESeq2 package in the R environment. Counts were normalised using the median-of-ratios method, and target genes related to lipid metabolism were selected and categorised into functional groups. The analysis compared the log2 estimated fold change between qPSC and the other groups: sPSC, 2dPSC, and 5dPSC.

### Quantification and statistical analysis

Data in the figures were presented either as box-and-whisker plots with the box representing the interquartile range, the line indicating the median, and whiskers showing the minimum and maximum values, or as bar charts showing the mean ± standard deviation (SD). Statistical analyses were performed using GraphPad software. Data distributions were assessed for normality using the Shapiro-Wilk and D’Agostino–Pearson tests. In cases of discrepancy, visual inspection (Q–Q plots, histograms) was used to support the decision. For datasets that followed a normal distribution, either one-way ANOVA with Sidak’s multiple comparisons test or unpaired t-tests with Welch’s correction were used. For non-normally distributed data, the non-parametric Kruskal-Wallis test with Dunn’s post hoc test was applied. A p-value of 0.05 was set as the threshold for statistical significance, with adjustments for multiple comparisons made as appropriate. The statistical tests used are specified in each figure legend, and statistical significance is indicated by asterisks: *p < 0.05, **p < 0.01, and ***p < 0.001.

### Retrieval of transcriptomic data from the human protein atlas

To analyse gene expression patterns in pancreatic cell types, single-cell transcriptomic data were retrieved from the Human Protein Atlas (HPA) database (proteinatlas.org) under the Creative Commons Attribution-ShareAlike 4.0 International License. Specifically, data were accessed from the Tabula Sapiens Consortium molecular cell atlas [17] and the Human Protein Atlas reference dataset [18], both of which provide single-cell RNA sequencing (scRNA-seq) profiles of human exocrine and endocrine pancreatic cells. Expression levels for genes of interest were extracted and visualised as fractions of cells exhibiting expression above a threshold of ≥ 1 nTPM (normalised transcripts per million). Heat maps representing these expression levels were generated using numerical annotations to indicate relative abundance across different cell clusters. To ensure consistency in visual representation, colour codes for heat map generation were sourced from the Human Protein Atlas and extracted using imagecolourpicker.com.

### Language edition

Artificial intelligence tools, including large language models, were used to assist in language editing and improving the readability of the manuscript. The authors reviewed and approved all AI-assisted revisions.

## Results

### Controlled activation of pancreatic stellate cells: quiescent to myofibroblast phenotypes

**Fig. 1:**
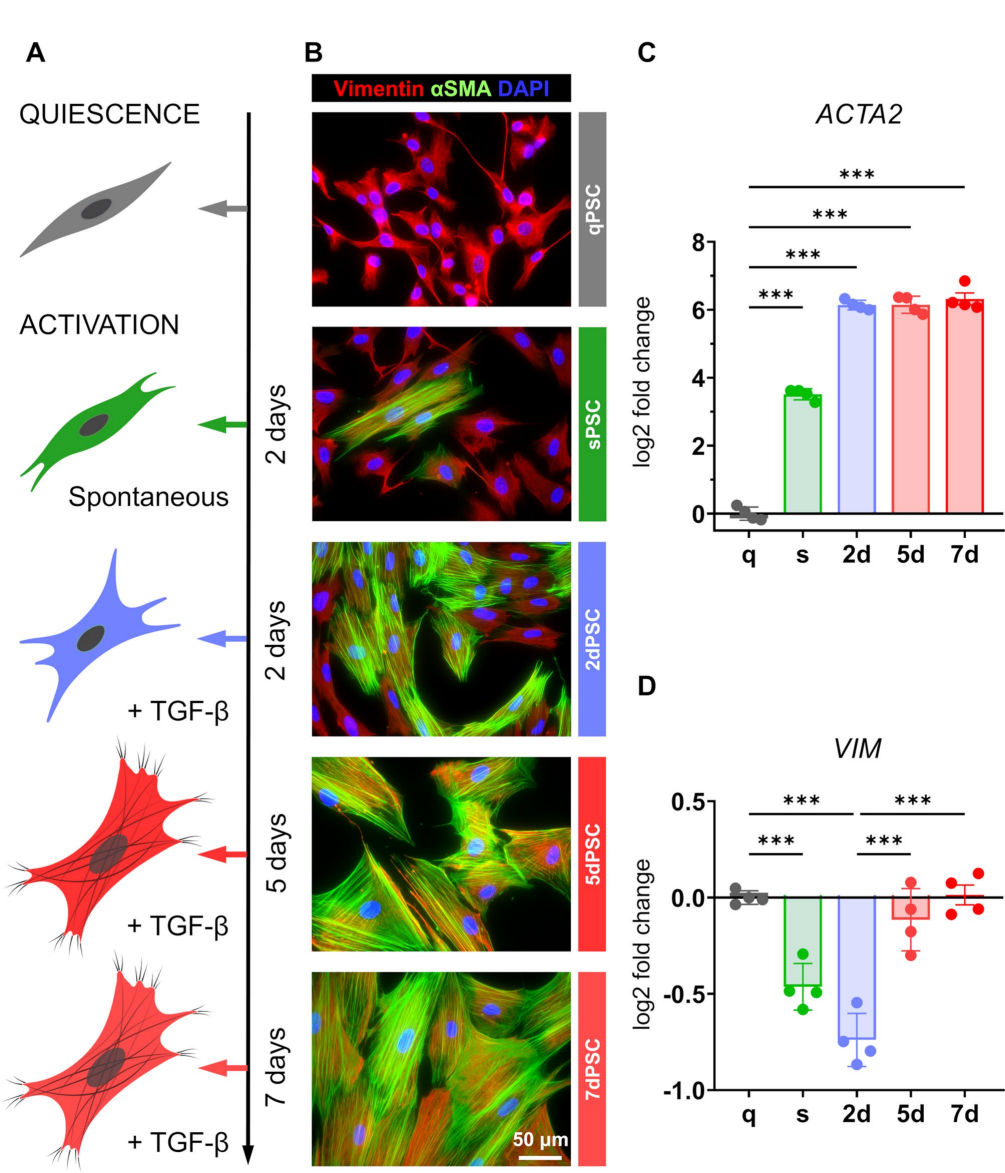
Activation of pancreatic stellate cells and cytoskeletal remodelling. (**A**) Schematic representation of PSC activation from quiescent (qPSC) to activated phenotypes, induced spontaneously for 2 days (sPSC) or with TGF-β treatment for 2 days (2dPSC) or 5–7 days (5/7dPSC). Created in BioRender. (**B**) Representative immunofluorescence images showing α-SMA (green) and vimentin (red) expression in PSC phenotypes, with DAPI nuclear staining (blue). The scale bar (50 μm) applies to all images. (**C**-**D**) Quantification of relative mRNA expression levels of *ACTA2* (encoding α-SMA) and *VIM* (encoding vimentin) across different PSC phenotypes. Data presented as log2 fold change (mean ± SD) relative to qPSC (number of independent biological replicates *N* = 4). Statistical comparisons were performed using one-way ANOVA followed by Sidak’s post hoc test for multiple comparisons. Statistical significance is indicated as: ***p < 0.001; results without a significance mark are non-significant (ns)

To explore the biophysical and biochemical alterations occurring during PSC activation, we used human cells in four phenotypes representing different activation stages (Fig. 1A): quiescent (qPSC), spontaneously activated (sPSC), and TGF-β-induced (5 ng/mL) for 2 days (2dPSC, corresponding to early activation) and 5 or 7 days (5dPSC or 7dPSC, respectively, both corresponding to late activation).

Activated PSCs are characterised by α-SMA expression, a myofibroblast marker [4]. Immunohistochemical staining shows α-SMA (green) and vimentin (red), typical for mesenchymal cells (Fig. 1B); mRNA expression is shown in Fig. 1C and D. qPSCs exhibit spindle-shaped, fibroblast-like morphology with thin, elongated bodies and minimal cytoplasmic extensions, showing little to no α-SMA expression. In contrast, sPSCs display mixed fibroblast and myofibroblast features with limited α-SMA expression (Fig. 1B).

TGF-β activated PSCs (2dPSC and 5/7dPSC) undergo marked morphological changes indicative of a phenotypic transition towards myofibroblasts. 2dPSCs are larger, and more elongated, exhibiting early cytoskeletal reorganisation with extended cytoplasmic projections (Fig. 1B). At late activation (5dPSC and 7dPSC), the cells exhibit a fully developed myofibroblast-like morphology with flattened, elongated shapes and extensive cytoplasmic spread. As we demonstrated in our previous work, α-SMA expression rises from ~ 60% in 2dPSCs (with visible actin fibres) to 100% in 5/7dPSCs, indicating complete myofibroblast maturation [12]. While α-SMA transcript (*ACTA2*) levels are particularly high in all activated phenotypes (Fig. 1C), vimentin expression shows a modest decrease during spontaneous and early activation, returning to baseline levels at the late activation stages (Fig. 1D). Since 5dPSCs and 7dPSCs both represent the late stage of PSC activation and exhibit comparable levels of α-SMA and vimentin expression, we considered them biologically equivalent. To maintain internal consistency within each experimental technique, only one of these time points was selected for each analysis.

### Cellular stiffness and topography in PSC phenotypes

To quantify the biophysical alterations associated with PSC activation, atomic force microscopy (AFM) was employed as a powerful tool for measuring cellular stiffness and morphology. AFM utilises a sharp cantilever tip to interact with the cell surface, generating force-distance curves that provide data on the cell’s mechanical properties, particularly the Young’s modulus (elastic modulus). The AFM technique enables the creation of high-resolution maps of stiffness variations, revealing the mechanical characteristics of individual cells.

**Fig. 2:**
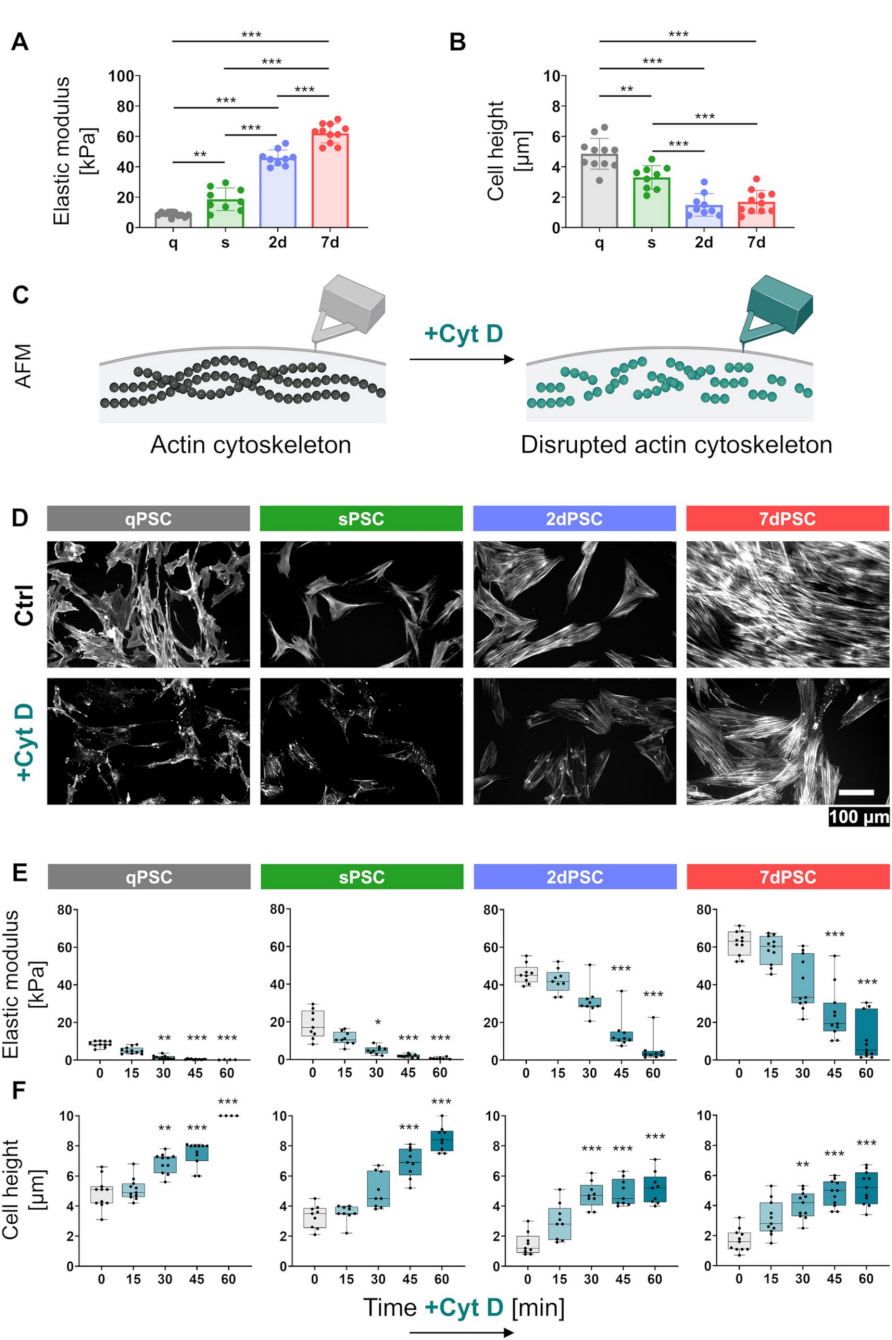
Biomechanical alterations in PSCs measured by AFM. (**A**) Young’s modulus (elasticity) measurements of PSCs at different activation states, showing a progressive increase in cellular stiffness from qPSC to 7dPSC (qPSC: *n* = 11, sPSC: *n* = 9, 2dPSC: *n* = 9, 7dPSC: *n* = 11). The same data are shown in Fig. 2E (grey boxes). Statistical comparisons were performed using one-way ANOVA with Sidak’s multiple comparisons. Statistical significance is indicated as: **p < 0.01, ***p < 0.001. (**B**) Changes in cell height during activation, indicating significant flattening (qPSC: *n* = 11, sPSC: *n* = 9, 2dPSC: *n* = 9, 7dPSC: *n* = 11). The same data are shown in Fig. 2F (grey boxes). Statistical comparisons were performed using one-way ANOVA with Sidak’s multiple comparisons. Statistical significance is indicated as: **p < 0.01, ***p < 0.001; results without a significance mark are non-significant (ns). (**C**) Schematic illustration of AFM experiments with and without cytochalasin D (Cyt D) treatment to disrupt the actin cytoskeleton. Created in BioRender. (**D**) Immunofluorescence images showing the effect of Cyt D on actin filaments in PSCs following 30 min of Cyt D treatment. The scale bar (100 μm) applies to all images. (**E**) Young’s modulus measurements in different PSC phenotypes following Cyt D treatment (50 µM) for 15, 30, 45 and 60 min. Statistical comparisons were performed using the non-parametric Kruskal–Wallis test with Dunn’s post hoc test. Statistical significance – compared to respective controls at 0 min – is indicated as: *p < 0.05, **p < 0.01, ***p < 0.001; results without a significance mark are non-significant (ns). (**F**) Cell height measurements in different PSC phenotypes following Cyt D treatment (50 µM) for 15, 30, 45 and 60 min. Data presented as mean ± SD. Statistical comparisons were performed using the non-parametric Kruskal –Wallis test with Dunn’s post hoc test. Statistical significance significance – compared to respective controls at 0 min – is indicated as: **p < 0.01, ***p < 0.001; results without a significance mark are non-significant (ns)

Elastic modulus analysis indicates that qPSCs exhibit relatively low stiffness, with an average modulus of 8.62 ± 1.55 kPa. Upon spontaneous activation (sPSCs), stiffness significantly increases to 18.62 ± 7.43 kPa, with further rises observed in two-day activated PSCs (2dPSC, 45.86 ± 5.23 kPa) and seven-day activated PSCs (7dPSC, 62.06 ± 6.37 kPa) (Fig. 2A). Additionally, the quiescent state corresponds to the greatest cell thickness (4.85 ± 1.02 μm), which decreases significantly upon activation (sPSC: 3.30 ± 0.77 μm; 2dPSC: 1.49 ± 0.74 μm) and remains stable in late activation stages (7dPSC: 1.69 ± 0.75 μm) (Fig. 2B).

To explore the contribution of the cytoskeleton to PSC mechanical properties, 50 µM cytochalasin D (Cyt D) was applied to disrupt actin polymerisation (schematic illustration in Fig. 2C). Fluorescence images illustrate the loss of actin filaments across all PSC phenotypes following 30 min of Cyt D treatment (Fig. 2D). Stiffness measurements indicate a time-dependent reduction in Young’s modulus after actin disruption, with an immediate decrease observed in qPSCs and sPSCs after 30 min (*p* = 0.001 and *p* = 0.0307, respectively). In contrast, in activated cells (2dPSC and 7dPSC), a significant decline occurs only at later time points (*p* < 0.001) (Fig. 2E). This delayed response in activated PSCs is likely attributable to their more stable actin stress fibres, which require prolonged Cyt D exposure for substantial depolymerisation.

Simultaneously with the reduction in stiffness, cell thickness progressively increased in all phenotypes following Cyt D treatment (Fig. 2F). The most pronounced thickening was observed after 45–60 min, with qPSCs displaying the highest expansion potential (> 10 μm, exceeding the detection capacity). In contrast, 2dPSCs and 7dPSCs exhibited a relatively constrained expansion capacity, suggesting that activated PSCs possess a structurally reinforced cytoskeleton that limits volume increase upon actin disruption.

### High-resolution quantitative imaging of PSC biomechanics

**Fig. 3:**
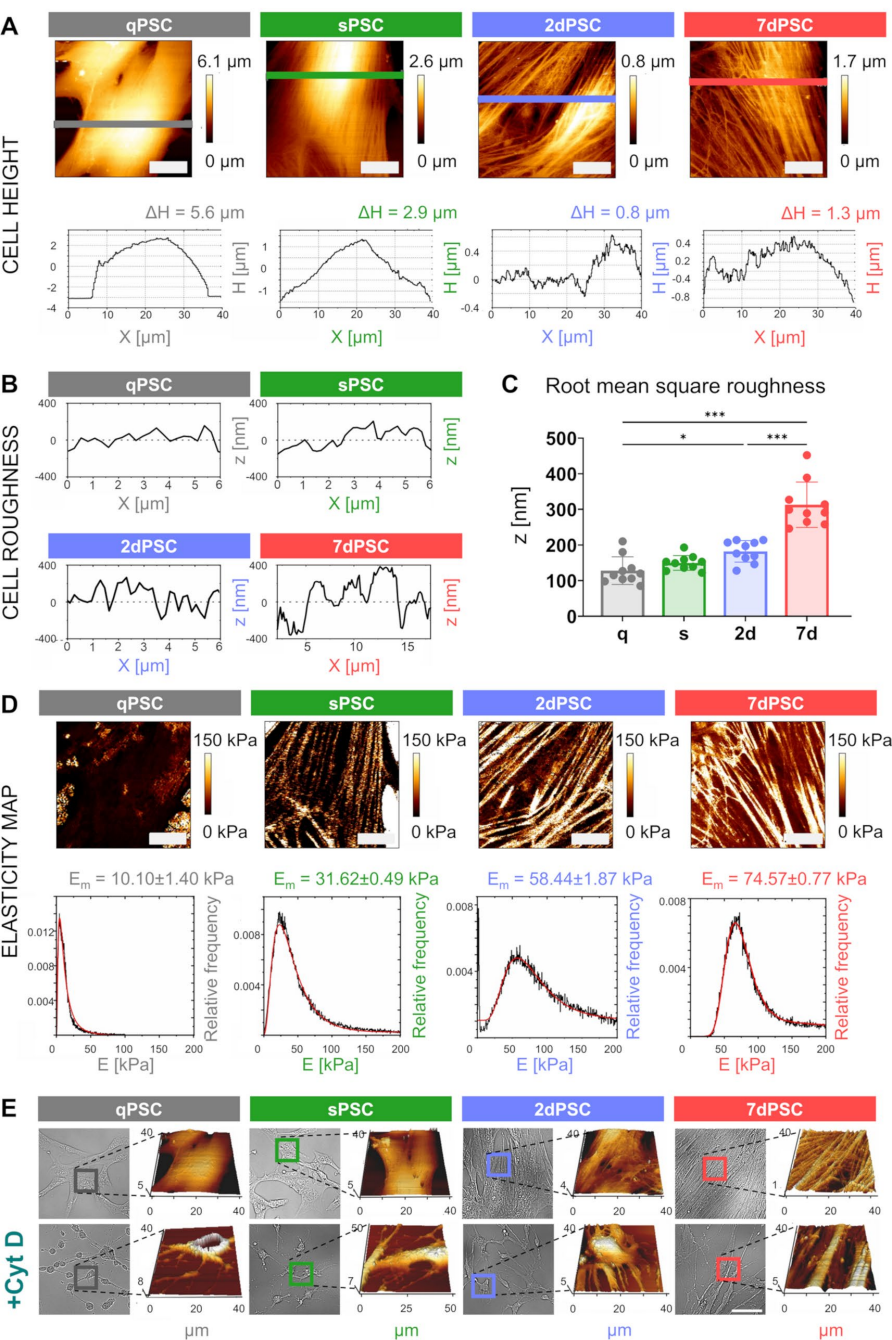
High-resolution AFM imaging of PSC topography and elasticity. (**A**) Representative topography maps of PSC phenotypes generated using AFM-based quantitative imaging mode (QI). Cell height (ΔH) is progressively reduced with activation. All scale bars: 10 μm. (**B**) Representative cell roughness maps derived from root mean square (RMS) roughness analysis, shown for each PSC phenotype (central cell area). (**C**) Quantification of RMS roughness values for each PSC phenotype (*n* = 10 per phenotype). Statistical comparisons were performed using one-way ANOVA followed by Sidak’s post hoc test for multiple comparisons. Significance is indicated as: *p < 0.05, ***p < 0.001; results without a significance mark are non-significant (ns). (**D**) Elasticity maps showing increased stiffness in activated PSCs. Histograms depict the relative frequency distribution of Young’s modulus values for each phenotype. The red line in each panel represents a log-normal fit to the distribution of Young’s modulus values. Scale bars: 10 μm. (**E**) AFM-QI analysis before and after Cyt D treatment, revealing morphological alterations and loss of cytoskeletal integrity in all PSC phenotypes. Scale bar: 50 μm (applies to all optical images)

To obtain a more precise and detailed characterisation of PSC topography and elasticity, AFM-based quantitative imaging (QI) mode was employed using a high-resolution conical probe with a 10 nm tip radius. Unlike standard force spectroscopy, QI mode enables high-resolution mapping of both topography (Fig. 3A-C) and mechanical stiffness (Fig. 3D), with enhanced sensitivity to subcellular structures. The use of a high-resolution conical probe facilitates the acquisition of deeper structural information, potentially revealing stiffness variations within lower cellular layers. These enhanced measurements provide a more precise assessment of cytoskeletal remodelling throughout PSC activation.

Representative topographical maps (Fig. 3A) provide a cross-sectional view of cell thickness across different PSC phenotypes, illustrating variations in cell height (ΔH), which represents the maximum thickness difference within each cell. Across all phenotypes, the greatest cell thickness is observed over the nuclear region. However, distinct differences in peripheral morphology are evident. In qPSCs, the height profile exhibits a semicircular shape, indicating that these cells retain considerable thickness even at the periphery. Upon spontaneous activation (sPSCs), the height profile shifts to a triangular shape, where the cell remains thickest over the nucleus but shows more pronounced flattening at the periphery. In 2dPSCs and 7dPSCs, extensive cell spreading is observed, with a substantial reduction in overall thickness, as seen in the representative examples in Fig. 3A, where ΔH reaches 0.8 μm and 1.3 μm, respectively. Notably, the TGF-β-activated PSCs exhibit increased height heterogeneity, likely reflecting the presence of an extensively remodelled actin cytoskeleton. This is demonstrated by RMS roughness analysis, calculated from surface cross-sections across the central regions of the cells, which quantifies the degree of local height variation. Figure 3B and C show representative surface profiles illustrating typical cross-sectional height variation and a quantitative comparison of RMS roughness across conditions, respectively. The RMS values demonstrate a modest increase in 2dPSCs and a pronounced elevation in 7dPSCs, indicating progressive surface irregularity corresponding to underlying actin stress fibres and cytoskeletal rearrangements that contribute to the mechanical stability and contractility of activated PSCs.

Elastic modulus maps (Fig. 3D) illustrate the progressive stiffening of PSCs during activation and reveal shifts in mechanical heterogeneity. Each map is accompanied by a histogram illustrating the relative frequency distribution of Young’s modulus, reflecting cytoskeletal organisation at different activation stages. In qPSCs, the distribution is narrow and centred around lower values (10.10 ± 1.40 kPa), indicating a relatively uniform and soft cytoskeletal structure. In sPSC, stiffness increases (31.62 ± 0.49 kPa), and the distribution broadens, suggesting the formation of mechanically distinct regions due to minor cytoskeletal remodelling. In 2dPSCs, stiffness further rises (58.44 ± 1.87 kPa), and the distribution becomes highly heterogeneous, likely due to the emergence of stiff actin stress fibres interspersed with softer areas. By 7dPSC, stiffness peaks (74.57 ± 0.77 kPa), but the distribution narrows slightly, which is consistent with a more mechanically stabilised yet reinforced cytoskeletal structure.

Figure 3E presents AFM-QI analysis of fixed PSCs before (upper panel) and after 60 min of Cyt D treatment (lower panel), corresponding to conditions shown in Fig. 2F. Each phenotype is represented by an optical image alongside a high-resolution topographical map (µm) acquired in QI mode. The images reveal distinct morphological alterations following actin cytoskeleton disruption, with cells becoming less structured and exhibiting increased height variability (brighter colours).

### Biochemical characterisation of PSC phenotypes using Raman spectroscopy

Raman spectroscopy (RS) has emerged as a powerful, non-invasive, label-free technique for studying molecular changes in various biological processes. By analysing vibrational signatures, it enables the investigation of cellular components such as proteins, lipids, and nucleic acids in their native state, without the need for invasive procedures or exogenous probes.

**Fig. 4:**
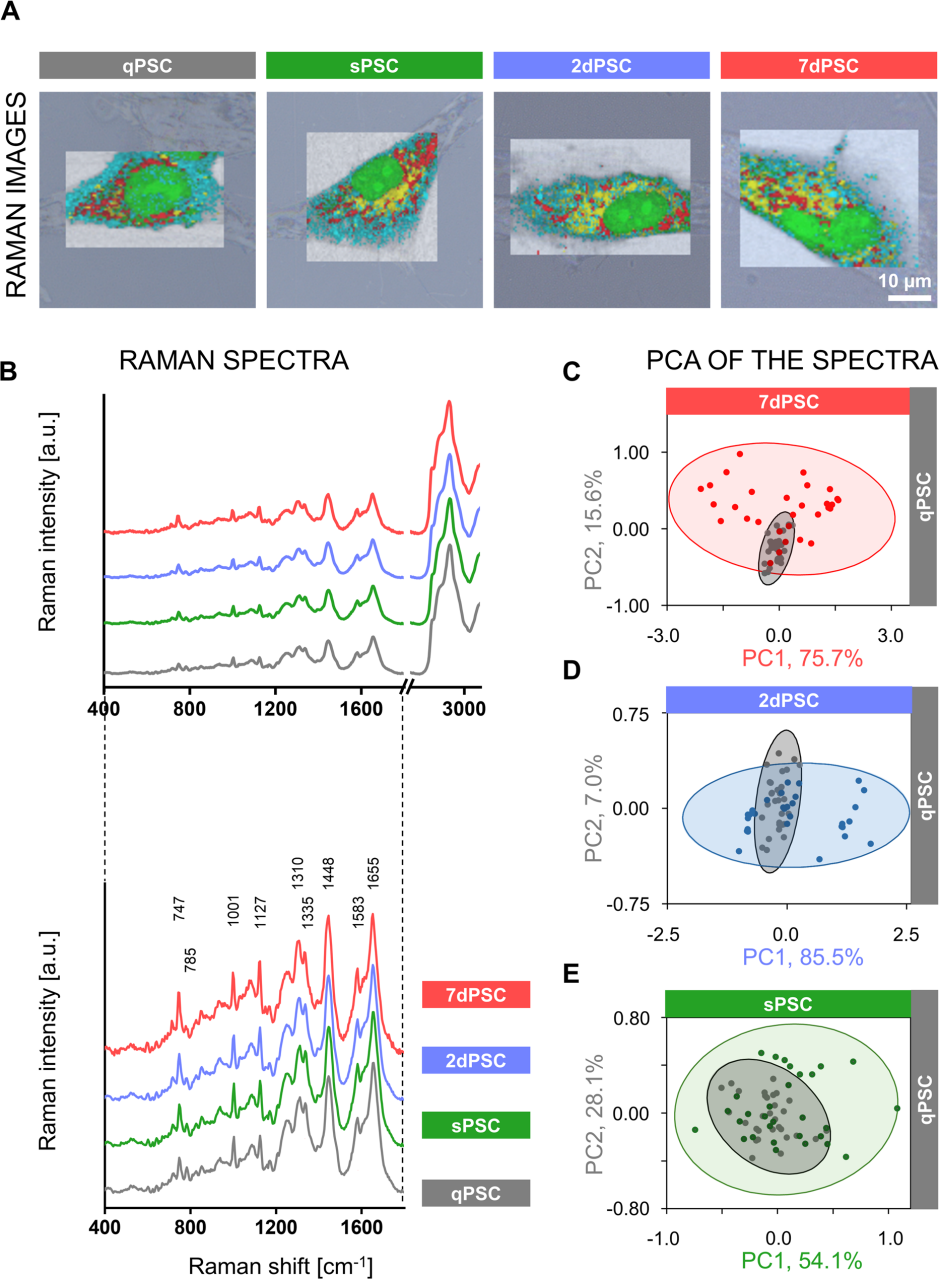
Biochemical characterisation of PSC phenotypes using Raman spectroscopy. (**A**) Representative Raman microscopy images of four PSC phenotypes. Raman images were generated using true component analysis (TCA) and overlaid on brightfield images. The colour-coding corresponds to: green – nucleic acids, red – cytochrome, yellow – lipids, and cyan – cytoplasm. Raman images were acquired using 532 nm excitation. Scale bar (10 μm) applies to all images shown. (**B**) Average Raman spectra of different PSC phenotypes: qPSC (grey), sPSC (green), 2dPSC (blue), and 7dPSC (red), shown in the range of 400–3200 cm⁻¹, with a magnified view of the 400–1800 cm⁻¹ region. (**C**-**E**) Principal component analysis (PCA) of Raman spectra, illustrating spectral differences between PSC phenotypes. Colours represent different phenotypes: qPSC (grey), sPSC (green), 2dPSC (blue), and 7dPSC (red). Ellipses indicate 95% confidence intervals

Figure 4A shows exemplary Raman images of representative PSC phenotypes (qPSC, sPSC, 2dPSC, 7dPSC), collected using a 532 nm excitation laser, allowing detailed molecular mapping of the cells’ biochemical composition, constructed using true component analysis (TCA) and overlayed on the brightfield images of the cells. The images provide a clear visualisation of the different biochemical components present in each phenotype (colour-coding: green for nucleic acids, red for cytochrome, yellow for lipids, and cyan for the cytoplasm).

The average Raman spectra for the four PSC phenotypes are depicted in Fig. 4B (lower panel and a zoomed-in view of the Raman spectra between 400 cm⁻¹ and 1800 cm⁻¹ in the lower panel). The Raman shifts provide information about the biochemical composition of the cells. For the spectra acquired with the 532 nm excitation line, several bands are dominated by the resonance enhancement of cytochrome signals. In particular, the bands at 747, 1127, 1310, and 1583 cm⁻¹ can be attributed to different vibrational modes of the porphyrin ring within cytochrome molecules [19, 20]. The high intensity of these bands reflects the resonance effect specific to this excitation condition. Additionally, the band at 785 cm⁻¹ is characteristic of nucleic acids, primarily associated with the symmetric stretching of the phosphate backbone [21, 22]. The band at 1001 cm⁻¹ is commonly assigned to the phenylalanine residue in proteins and can serve as a marker of protein content. The 1448 cm⁻¹ band, corresponding to CH₂ and CH₃ bending vibrations, arises from both lipids and proteins, reflecting the presence of these groups in cellular structures. Finally, the 1655 cm⁻¹ band, representing the amide I vibration, provides information about the secondary structure of proteins, especially α-helices [21, 23].

Principal component analysis (PCA) reveals distinct clustering between the different activated phenotypes compared to qPSCs (Figs. 4C-E). The most pronounced biochemical divergence is observed between qPSCs and 7dPSCs, the two most extreme phenotypes (Fig. 4C). When comparing qPSCs and 2dPSCs, the qPSCs display a narrower distribution, indicating a more homogeneous biochemical profile, whereas the broader distribution of 2dPSCs may reflect the variability introduced by TGF-β activation (Fig. 4D). This variability aligns with observations that only about 60% of 2dPSCs express α-SMA, with the remaining 40% retaining a profile closer to qPSCs or sPSCs [12]. Even spontaneous activation on its own induces some biochemical variability, as shown by the larger 95% confidence ellipse for sPSCs (Fig. 4E).

**Fig. 5:**
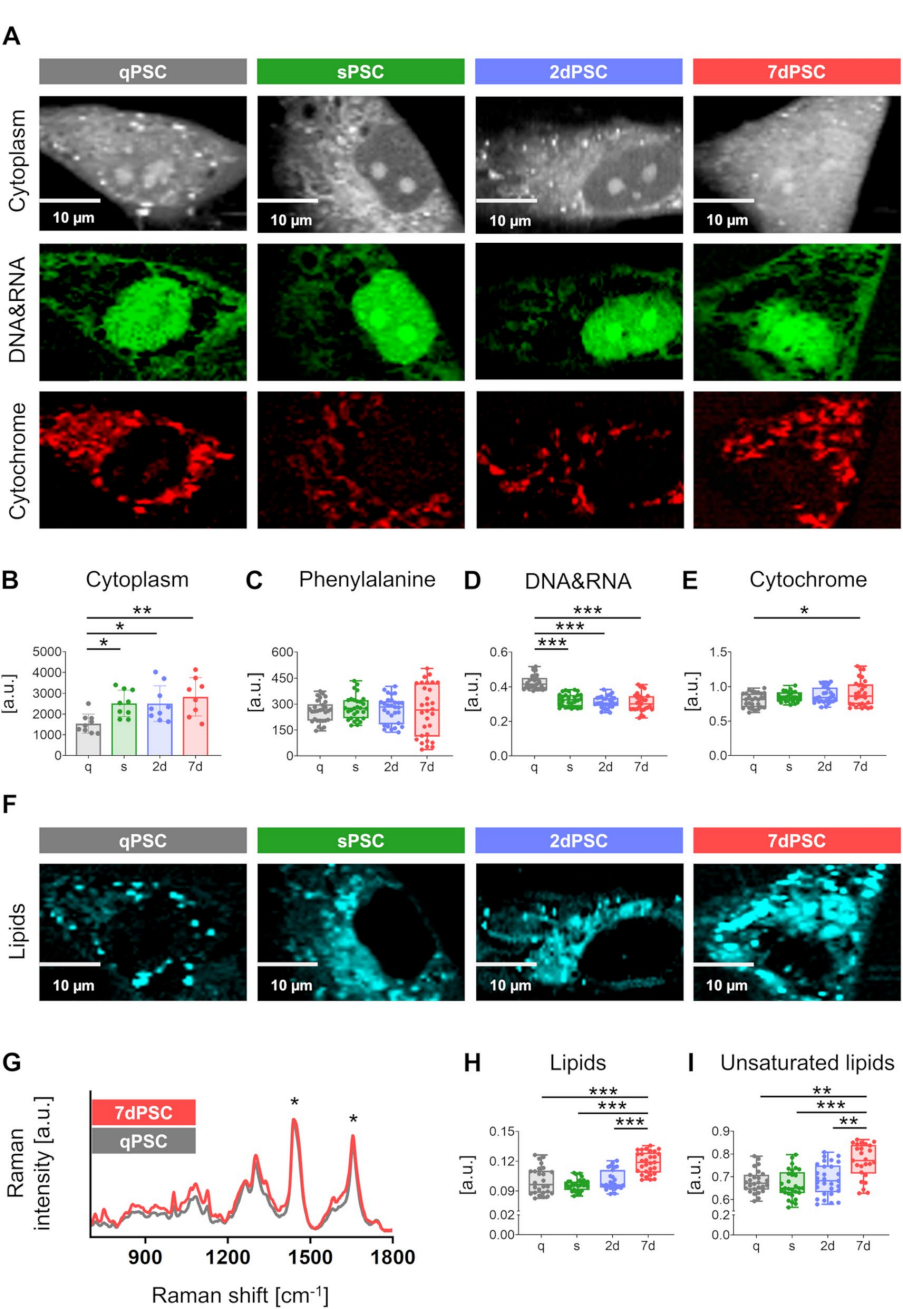
Molecular composition of PSCs determined by Raman imaging. (**A**) Representative Raman images illustrating the spatial distribution of cytoplasm (grey), cytochrome (red), nucleic acids (green), and lipids (cyan) in quiescent (qPSC), spontaneously activated (sPSC), and TGF-β-induced PSCs activated for 2 days (2dPSC) and 7 days (7dPSC). Scale bars apply to all images. (**B**) Semi-quantitative analysis of cell size based on calculated area of the cytoplasm (number of pixels comprising signal from cytoplasm); *N* = 3 independent experiments (biological replicates); n = total number of cells measured across replicates (qPSC, sPSC, 2dPSC: *n* = 9; 7dPSC: *n* = 8). Statistical comparisons were performed using one-way ANOVA with Sidak’s multiple comparisons. Statistical significance is indicated by *p < 0.05, **p < 0.01; results without a significance mark are non-significant (ns). (**C**–**E**) Semi-quantitative analysis based on the ratios of integrated intensities of selected Raman bands corresponding to: (**C**) phenylalanine (*N* = 3 independent experiments; qPSC: *n* = 30, sPSC: *n* = 30, 2dPSC: *n* = 30, 7dPSC: *n* = 30), (**D**) nucleic acids (*N* = 3; qPSC: *n* = 29, sPSC: *n* = 28, 2dPSC: *n* = 30, 7dPSC: *n* = 29), and (**E**) cytochrome (*N* = 3; qPSC: *n* = 28, sPSC: *n* = 27, 2dPSC: *n* = 28, 7dPSC: *n* = 29). Integrated intensity was normalised to the phenylalanine band at 1001 cm⁻¹. Statistical comparisons were performed using the non-parametric Kruskal–Wallis test with Dunn’s post hoc test (**C**–**D**) or one-way ANOVA with Sidak’s multiple comparisons (**E**). Statistical significance indicated as: *p < 0.05, ***p < 0.001; results without a significance mark are non-significant (ns). (**F**) Representative Raman images illustrating the distribution of lipids (cyan) in qPSC, sPSC, 2dPSC, and 7dPSC. (**G**) Representative Raman spectra in the range of 700–1800 cm⁻¹, showing differences between more (red) and less (grey) saturated lipids in qPSC and 7dPSC. Statistical significance indicated as: *p < 0.05. (**H**) Semi-quantitative analysis of lipid content based on the integrated intensity ratios of lipid-related Raman bands (*N* = 3 independent experiments; qPSC: *n* = 26, sPSC: *n* = 29, 2dPSC: *n* = 27, 7dPSC: *n* = 29). Intensities were normalised to the phenylalanine band at 1001 cm⁻¹. Statistical comparisons were performed using the non-parametric Kruskal–Wallis test with Dunn’s post hoc test. Statistical significance indicated as: ***p < 0.001; results without a significance mark are non-significant (ns). (**I**) Semi-quantitative analysis of lipid saturation degree based on the ratio of saturated (1448 cm⁻¹) to unsaturated (1655 cm⁻¹) lipids among the analysed PSC phenotypes (*N* = 3 independent experiments; qPSC: *n* = 26, sPSC: *n* = 29, 2dPSC: *n* = 29, 7dPSC: *n* = 25). Statistical comparisons were performed using the non-parametric Kruskal–Wallis test with Dunn’s post hoc test. Statistical significance indicated as: **p < 0.01, ***p < 0.001; results without a significance mark are non-significant (ns)

Representative Raman integration images show the distribution of specific cellular components – cytoplasm (grey), nucleic acids (green), and cytochrome (red) – across qPSCs, sPSCs, 2dPSCs and 7dPSCs (Fig. 5A). An expansion of the cytoplasmic area was observed during PSC activation, starting in sPSCs and becoming more pronounced in 7dPSCs (Fig. 5B).

Importantly, the mean phenylalanine (Phe) levels remain consistent across all groups, with only slightly greater variability in the 7dPSC group (Fig. 5C). This stability suggests that Phe levels are unaffected by the phenotype transition, even as activated cells increase synthesis of cytoskeletal and ECM components. Normalisation to the distinct Raman band of phenylalanine (Phe, around 1001 cm⁻¹) is commonly applied to account for variability in laser intensity and other measurement conditions.

Interestingly, a significant decrease in nucleic acid (blue) signal was evident in sPSCs, 2dPSCs, and 7dPSCs compared to qPSCs, suggesting alterations in chromatin organisation or transcriptional activity (Fig. 5D). Cytochrome (red) levels showed a gradual increase upon activation, reaching statistical significance only in 7dPSCs, indicating heightened metabolic activity at later activation stages (Fig. 5E).

Representative Raman integration images for lipids (cyan) illustrate lipid distribution across PSC phenotypes at different activation stages (Fig. 5F). Figure 5G presents Raman spectra of qPSCs and 7dPSCs, highlighting distinct lipid-associated bands. A notable difference is observed in the ~ 1445 cm⁻¹ band, corresponding to CH₂ bending vibrations, indicative of lipid chain organisation. Additionally, the ~ 1655–1670 cm⁻¹ band, associated with C = C stretching in unsaturated lipids, is more pronounced in 7dPSCs, suggesting changes in lipid composition upon late activation. A statistically significant increase in lipid content was observed, particularly in 7dPSCs compared to qPSCs (Fig. 5H), which is also clearly illustrated in the rightmost image of Fig. 5F. Additionally, the proportion of unsaturated lipids increased during activation (Fig. 5I), highlighting lipid composition changes associated with PSC activation.

### Lipid composition and related gene expression during PSC activation

**Fig. 6:**
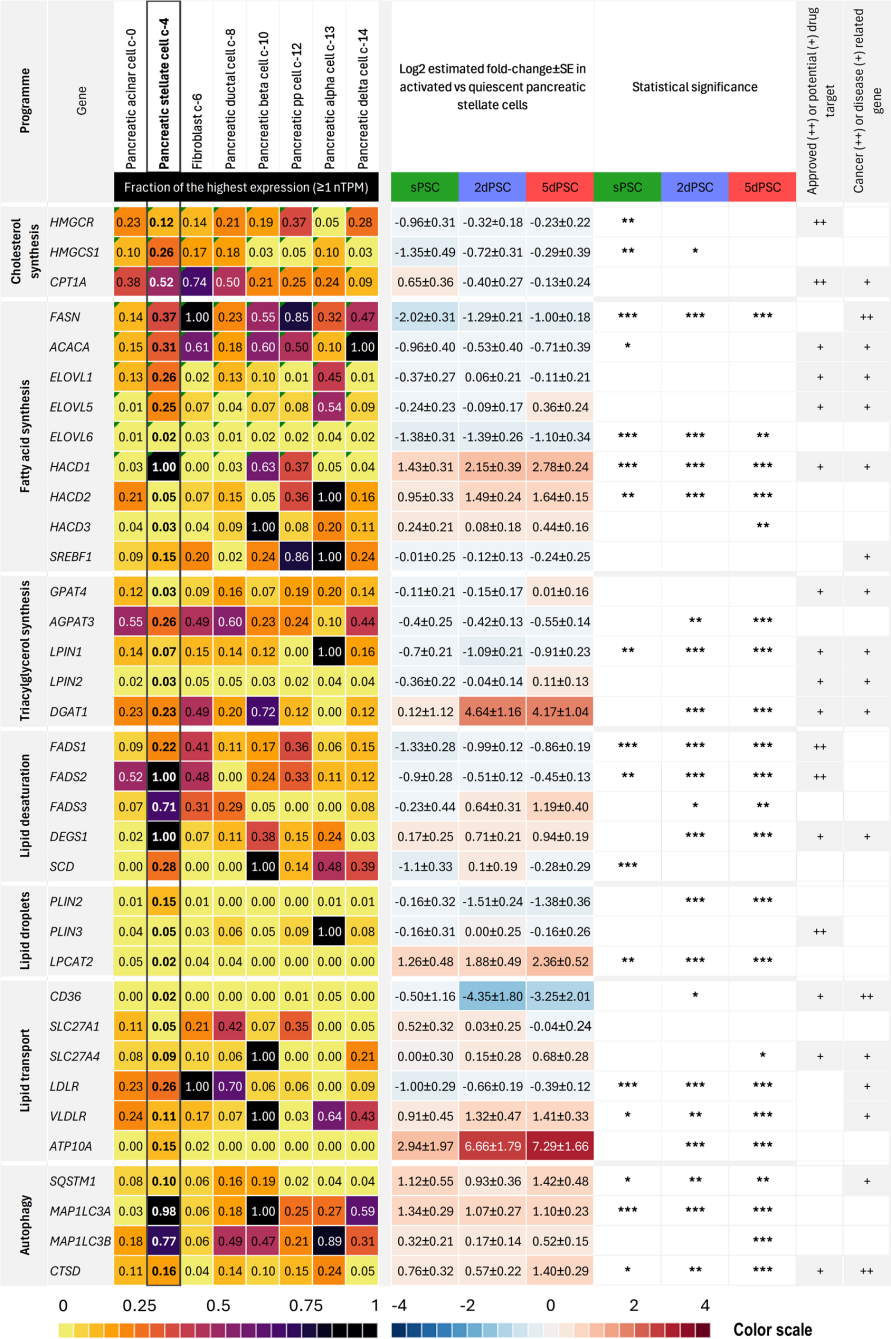
Transcriptomic analysis of lipid metabolism in PSC activation. **Left**: Expression profiles of genes involved in cholesterol synthesis, fatty acid metabolism, lipid desaturation, lipid droplet formation, and autophagy in PSCs compared to other pancreatic cell types (retrieved from the Human Protein Atlas database: proteinatlas.org). **Middle**: Differential gene expression analysis in activated PSCs (sPSC, 2dPSC, 5dPSC) relative to quiescent PSCs, highlighting metabolic reprogramming. Functional categorisation of significantly upregulated and downregulated genes. Data shown as log2 fold change (mean ± SE), with statistical significance indicated as: *p < 0.05, **p < 0.01, ***p < 0.001; the absence of an asterisk indicates lack of statistical significance (ns). **Right**: Indicated approved or potential drug targets for reference purposes [17]

To further elucidate the shifts in lipid content, we analysed the expression of selected genes involved in cholesterol synthesis, fatty acid synthesis, triacylglycerol (TAG) synthesis, lipid desaturation, lipid droplet regulation, lipid transport, and autophagy (Fig. 6). These genes were chosen due to their key roles in these metabolic pathways. First, we used publicly available data from the Human Protein Atlas to compare their expression levels across different pancreatic cell types, including acinar cells, stellate cells, fibroblasts, ductal cells, beta, PP, alpha, and delta cells (Fig. 6, left) [17, 18]. Next, we analysed transcriptomic data, comparing the expression changes of these genes in sPSCs, 2dPSCs, and 5dPSCs relative to qPSCs (Fig. 6, middle). Finally, we investigated and clearly indicated which gene products are approved or potential drug targets and whether they are associated with cancer or other diseases (Fig. 6, right).

Interestingly, cholesterol biosynthesis genes, which are expressed at moderate-to-high levels in PSCs compared to other pancreatic cell types, were notably downregulated in activated phenotypes. Specifically, the expression of *HMGCR* (3-hydroxy-3-methylglutaryl-CoA reductase) and *HMGCS1* (3-hydroxy-3-methylglutaryl-CoA synthase 1), key enzymes in the mevalonate pathway, was reduced, suggesting a decreased cholesterol demand, likely due to reduced cell proliferation and a shift towards TAG accumulation. Similarly, fatty acid synthesis genes *FASN* (fatty acid synthase) and *ACACA* (acetyl-CoA carboxylase alpha) were downregulated, indicating suppression of *de novo* lipogenesis and reduced production of saturated fatty acids. In contrast, elongation genes *HACD1* (3-hydroxyacyl-CoA dehydratase 1) and *HACD2* (3-hydroxyacyl-CoA dehydratase 2) were significantly upregulated. Notably, *HACD1* expression is the highest in PSCs among pancreatic cell types and was strongly induced in activated cells (log2 fold change 2.78 in 5dPSCs, Fig. 6), suggesting enhanced elongation of existing fatty acids to support membrane remodelling.

We observed a shift in the conventional glycerol-3-phosphate pathway. Downregulation of *GPAT4* (glycerol-3-phosphate acyltransferase 4), *AGPAT3* (1-acylglycerol-3-phosphate O-acyltransferase 3), and *LPIN1* (lipin 1) – enzymes responsible for the initial steps of TAG synthesis – alongside strong upregulation of *DGAT1* (diacylglycerol O-acyltransferase 1), lipid droplet accumulation, and increased autophagy suggests an alternative source of substrates for TAG synthesis.

Lipid desaturation genes were highly expressed in PSCs compared to other pancreatic cell types, particularly *FADS2* (fatty acid desaturase 2), *FADS3* (fatty acid desaturase 3), and *DEGS1* (delta 4-desaturase, sphingolipid 1), with the latter two showing significant upregulation in 2dPSCs and 5dPSCs. High expression of *FADS2* and *FADS3* suggests enhanced fatty acid modification, potentially supplying specific polyunsaturated fatty acids for membrane remodelling or lipid droplet storage. Additionally, increased *DEGS1* expression, whose product converts dihydroceramide to ceramide, indicates heightened sphingolipid metabolism, which may be linked to stress signalling and autophagy.

The regulation of lipid droplets also changed during activation. *PLIN2* (perilipin 2), which stabilises small lipid droplets, was downregulated, favouring fewer but larger droplets. Conversely, *LPCAT2* (lysophosphatidylcholine acyltransferase 2), involved in phospholipid remodelling, was upregulated (log2 fold change 2.36 at 5dPSC), enhancing lipid droplet capacity. *LPCAT2* is known to contribute to phospholipid remodelling, potentially leading to increased diacylglycerol (DAG) release [24]. This suggests that elevated *LPCAT2* activity may indirectly provide DAG for TAG synthesis, further supporting lipid droplet accumulation.

Genes related to lipid transport exhibited significant changes. *CD36* (CD36 molecule, thrombospondin receptor), which mediates external fatty acid uptake [25], was markedly downregulated (log2 fold change − 4.35 at 2d, -3.25 at 5d), whereas *SLC27A4* (solute carrier family 27 member 4) and *VLDLR* (very low-density lipoprotein receptor) were upregulated, suggesting that activated PSCs shift metabolism towards internal lipid reserves and membrane remodelling. Additionally, *ATP10A* (ATPase phospholipid transporting 10 A), a phospholipid flippase, exhibited profound upregulation (log2 fold change 7.29 at 5dPSC), reflecting active membrane remodelling.

Autophagy-related genes, including *SQSTM1* (sequestosome 1, also known as p62), *MAP1LC3A* (microtubule-associated protein 1 light chain 3 alpha), *MAP1LC3B* (microtubule-associated protein 1 light chain 3 beta), and *CTSD* (cathepsin D), not only exhibited relatively high expression in PSCs (compared to other pancreatic cell types) but were also significantly upregulated in activated cells. These changes likely facilitate lipid droplet recycling, providing fatty acids for energy production and membrane remodelling, emphasising the crucial role of autophagy in lipid turnover during PSC activation.

## Discussion

In this study, we investigated the mechanical and biochemical changes occurring during the activation of PSCs. Our findings revealed that activated PSCs become significantly flattened and exhibit increased stiffness, as shown by atomic force microscopy. These mechanical adaptations are primarily driven by cytoskeletal reorganisation, notably the formation of actin stress fibres, which are characteristic of the myofibroblast-like phenotype and may reflect an integrated response to mechanical and paracrine cues.

Previous studies have employed AFM to assess the stiffness of pancreatic tumour tissues and co-culture systems, revealing that stellate cell activation correlates with increased tissue rigidity due to collagen deposition [26]. Our findings extend this understanding by showing that individual PSCs themselves become markedly stiffer and more flattened upon activation (Fig. 2A and B). This phenomenon is closely linked to the upregulation of α-SMA, a hallmark of the contractile, myofibroblast-like phenotype and a prognostic indicator in pancreatic cancer [27]. The recovery of baseline mechanical characteristics upon cytoskeletal disruption by Cyt D provides strong evidence that the actin cytoskeleton is the principal driver of activation-related mechanical stiffening and flattening (Fig. 2E and F). In addition to global changes in stiffness and shape, we also observed increased microscale surface irregularity in activated PSCs, as demonstrated by elevated RMS roughness values (Fig. 3B and C). This topographical heterogeneity likely reflects cytoskeletal remodelling and may contribute to altered mechanical interactions with the surrounding matrix or neighbouring cells. Mechanical cues, such as elevated interstitial pressure and matrix stiffening, are increasingly recognised as instructive signals that drive PSC activation. The involvement of TRPC1 ion channels in this process highlights their role in mechanotransduction, translating extracellular mechanical stimuli into intracellular signalling events that regulate cytoskeletal dynamics and cell contractility [28]. These findings suggest that PSCs not only respond to mechanical forces but also participate actively in shaping the biomechanical signalling landscape of the fibrotic and tumour-associated microenvironment.

Consistent with the AFM results, Raman spectroscopy revealed an increased cellular surface area in activated PSCs (Fig. 5B), reflecting both morphological and structural changes. The elevated cytochrome signal indicates enhanced metabolic activity, which is expected given the increased production and secretion of extracellular matrix (ECM) components by activated PSCs. Interestingly, despite this heightened biosynthetic activity, the relative nucleic acid signal appeared reduced (Fig. 5D). This decrease is likely attributable to chromatin remodelling, where DNA adopts a more open, euchromatic state to facilitate transcriptional upregulation [29]. Less condensed chromatin scatters light less efficiently than heterochromatin, resulting in lower Raman signal intensity despite unchanged or elevated nucleic acid content [30, 31]. In addition, mRNA and ribosomal RNA may be redistributed to the cytoplasm to support protein synthesis, further reducing the nuclear nucleic acid signal during Raman imaging [22, 32].

In their quiescent state, PSCs store vitamin A in cytoplasmic lipid droplets enriched in retinyl esters, triacylglycerols, and other neutral lipids [1, 33]. Upon activation, these lipid stores are depleted, marking the transition to a myofibroblast-like phenotype [4, 34]. In our study, PSCs were cultured without retinoid supplementation, thereby limiting droplet formation typically seen in retinoid-rich conditions [4]. Notably, activated PSCs undergo extensive lipid metabolic remodelling, including increased synthesis of phosphatidylcholines and bioactive lipids such as lysophosphatidic acid (LPA), a potent signalling molecule known to promote tumour cell proliferation, migration, and survival [35]. Clearly, metabolic rewiring in activated PSCs is not merely a reflection of altered energy storage, but may actively contribute to paracrine signalling processes within the tumour and fibrotic microenvironment. The production of lipid-derived messengers such as LPA may facilitate stromal–epithelial and stromal–vascular communication, further amplifying pathological responses in pancreatic disease.

Although we did not establish a direct mechanistic link between cytoskeletal changes and lipid metabolism, the parallel emergence of mechanical and metabolic alterations during PSC activation suggests the possibility of coordinated adaptation. Changes in lipid composition – particularly increased unsaturation and phospholipid remodelling – may influence membrane properties such as curvature and fluidity, which in turn could modulate cytoskeletal organisation or cellular mechanics indirectly. While this remains speculative, the synchrony of these processes raises intriguing questions about potential crosstalk between mechanical and metabolic reprogramming in activated PSCs.

While early activation did not markedly alter the lipid profile of 2dPSCs, RS revealed a significant increase in lipid content and a higher degree of lipid unsaturation in 7dPSCs compared to quiescent cells. The Protein Atlas indicates that genes involved in lipid desaturation are particularly highly expressed in PSCs compared to other pancreatic cell types (Fig. 6). Transcriptomic analyses show further upregulation of genes involved in fatty acid elongation and desaturation, such as *FADS3* and *DEGS1*, while canonical desaturases *FADS1* and *FADS2* were downregulated (Fig. 6). These changes suggest a qualitative shift in unsaturated fatty acid composition, which may influence membrane properties such as fluidity, curvature, and the organisation of lipid microdomains involved in receptor signalling [36]. Moreover, modifications in membrane lipid composition are known to affect vesicle trafficking, cytokine release, and receptor localisation, all of which are critical for intercellular communication [37].

Simultaneously, activated PSCs downregulate genes involved in cholesterol (mevalonate pathway), fatty acid (*FASN*, *ACACA*), and TAG synthesis (*AGPAT3*, *LPIN1*), indicating a shift away from *de novo* lipogenesis. Instead, they increase *LPCAT2* expression, which facilitates membrane phospholipid remodelling, likely generating diacylglycerol (DAG) as a substrate for *DGAT1*, which is very significantly upregulated. Concurrently, enhanced autophagy (*SQSTM1*, *MAP1LC3A*, *MAP1LC3B*, *CTSD*) provides additional lipid sources through the degradation of internal cellular organelles and structures [38, 39]. This is particularly interesting in light of previous findings showing that PSC-derived alanine, generated via autophagy, fuels pancreatic cancer cell metabolism [40]. Considering the profound lipid metabolic remodelling observed in activated PSCs, including lipid droplet loss and phospholipid synthesis, it is tempting to speculate that, beyond alanine secretion, altered lipid metabolism may directly supply lipid substrates to pancreatic cancer cells, further supporting the metabolic crosstalk within the tumour microenvironment.

Perilipins are proteins that coat the surface of lipid droplets, protecting them from enzymatic breakdown by lipases and playing a key role in regulating lipid storage and utilisation in cells [41]. Decreased perilipin-2 (*PLIN2*) expression may promote the formation of fewer but larger lipid droplets. Previous studies have shown that knocking out *PLIN2* in hepatocytes or myoblast cell lines leads to enlarged lipid droplets and increased TAG hydrolysis [42, 43]. Of note is that *LPCAT2*, discussed earlier in phospholipid remodelling, may also promote lipid droplet accumulation, as its expression has been shown to correlate with their content in colorectal cancer cells [44]. Furthermore, *SQSTM1* (p62) is particularly interesting in this context, as it may mediate lipid droplet recognition and targeting to autophagosomes, facilitating their degradation in lysosomes [45, 46]. It is possible that, in activated PSCs, smaller lipid droplets are preferentially degraded via lipophagy, while larger lipid droplets become more stable and resistant to this process. This model aligns with the observed high expression of *SQSTM1* and autophagy markers (*ATG10*, *MAP1LC3B*), the downregulation of *PLIN2*, increased expression of *LPCAT2* and the accumulation of lipid droplets. This metabolic remodelling likely contributes to the pronounced Raman signal observed for lipid bands in activated PSCs.

Interestingly, *CD36* expression declines, suggesting a reduced reliance on exogenous fatty acid uptake [25]. Instead, *VLDLR* is upregulated, indicating a shift towards utilising very-low-density lipoproteins (VLDL) as an alternative lipid source. Meanwhile, *LDLR* remains relatively high but decreases slightly, reinforcing a selective adaptation in lipid uptake. This metabolic reprogramming suggests that PSCs selectively use lipid sources that provide not only fatty acids but also bioactive lipids such as sphingolipids.

What is also interesting is a very significant increase in *ATP10A* expression (Fig. 6), which functions as a phospholipid flippase, maintaining plasma membrane asymmetry by translocating phosphatidylserine and phosphatidylethanolamine to the inner leaflet [47, 48]. It is also important to note that *ATP10A* is almost exclusively expressed in PSCs among pancreatic cell types (may also be present in pancreatic fibroblasts at a very low level), and could be a novel marker of PSCs in the pancreas. Its upregulation could reflect increased vesicular trafficking needed for ECM secretion. Redistribution of membrane phospholipids also influences membrane curvature [49–51], enabling cell spreading, migration, and contractility, consistent with the observed changes in cell stiffness and morphology in activated PSCs. Furthermore, phosphatidylserine externalisation is a hallmark of apoptosis, signalling cell death and promoting immune clearance [52]. High *ATP10A* expression in activated PSCs may help prevent phosphatidylserine exposure, thus protecting the cells from immune recognition and supporting their prolonged survival during chronic activation in fibrotic tissue.

The metabolic rewiring of activated PSCs also reveals promising therapeutic opportunities. Several genes altered during activation are approved or potential drug targets (Fig. 6), including *HMGCR*, *FADS1/2* and *CPT1A*, while others, such as *FASN*, *CD36*, and *CTSD*, are directly implicated in cancer-related processes [17]. Targeting these pathways could selectively modulate PSC physiology, attenuate fibrosis, and disrupt metabolic crosstalk with pancreatic cancer cells.

Overall, our findings suggest that during PSC activation, profound biophysical and biochemical reprogramming occurs, enabling these cells to meet the increased functional demands associated with their transition to myofibroblast-like state. AFM measurements revealed increased cellular stiffness and morphological changes driven by cytoskeletal reorganisation, supporting enhanced contractile capacity. Concurrently, RS provided insights into underlying molecular changes, including a shift in lipid metabolism away from *de novo* synthesis towards elongation, desaturation, and storage in fewer but larger lipid droplets. These adaptations collectively support the matrix-producing and secretory phenotype of activated PSCs. Importantly, the integration of mechanical and metabolic alterations may not only sustain PSC activation, but also modulate their signalling output positioning them as active mediators of stromal communication in the fibrotic and tumour microenvironment.

## Data Availability

The datasets generated and/or analysed during the current study are available from the corresponding author upon reasonable request.
